# Short-Term Effects of Pecavaptan on Weight and Congestion in Acute Heart Failure: The AVANTI Post-Hoc Analysis

**DOI:** 10.1016/j.jacadv.2026.102891

**Published:** 2026-07-22

**Authors:** Daan C.H. Ceelen, Geert H.D. Voordes, Lucija Cosic, Jozine M. ter Maaten, Steven R. Goldsmith, Daniel Burkhoff, Finn Gustafsson, Robert Frost, Kevin Duarte, Luca Monzo, Nicolas Girerd, Faiez Zannad, James E. Udelson, Adriaan A. Voors

**Affiliations:** a Department of Cardiology, University of Groningen, University Medical Centre Groningen, Groningen, The Netherlands; b Hennepin County Medical Center, University of Minnesota, Minneapolis, Minnesota, USA; c Cardiovascular Research Foundation, New York, New York, USA; d Department of Clinical Medicine, University of Copenhagen; e Bayer AG, Research and Early Development, Cardiovascular Precision Medicines, Wuppertal, Germany; f Bayer AG, Research and Development, Pharmaceuticals, TA Statistics 1 CNTH, Berlin, Germany; g CHRU de Nancy, Centre d'Investigation Clinique Plurithématique 1433, INSERM, Université de Lorraine, INSERM U1116 - DCAC, F-CRIN INI-CRCT (Cardiovascular and Renal Clinical Trialists), Nancy, France; h Division of Cardiology and the CardioVascular Centre, Tufts Medical Center, Boston, Massachusetts, USA

**Keywords:** congestion, diuretics, heart failure, vasopressin, weight

## Abstract

**Background:**

Pecavaptan, a dual vasopressin V1a/V2-receptor antagonist, did not enhance weight loss by day 30 in hospitalized heart failure (HF) patients. However, its short-term effects remain unclear.

**Objectives:**

The objective of the study was to evaluate pecavaptan’s effect on weight loss and congestion after 7 days.

**Methods:**

AVANTI was a double-blind, randomized, placebo-controlled trial in patients hospitalized with HF and incomplete decongestion (n = 482). In part A, patients received pecavaptan (30 mg) (n = 242) or placebo (n = 240) as adjunctive therapy for 30 days. In this post hoc analysis, baseline-adjusted differences in weight change and a composite congestion score (CCS) at day 7 were calculated using analysis of covariance. Individual CCS component effects were assessed using baseline-adjusted ordinal regression at day 7.

**Results:**

Patients had a median age of 70 years (IQR: 62.0-78.0) and 24% (118/482) were women. Pecavaptan resulted in greater weight loss vs placebo by day 7 (−1.43 kg vs −0.95 kg; difference −0.47 kg; 95% CI: −0.94 to −0.01; *P* = 0.044). Although the CCS was not significantly modified by pecavaptan at day 7 (*P* = 0.171), we observed a better resolution of pedal edema (adjusted OR: 0.63; 95% CI: 0.42-0.95; nominal *P* = 0.026) and reduced thoracic fluid content (difference: −1.43%; 95% CI: −2.57 to −0.28; nominal *P* = 0.015) at day 7. These effects were limited and no longer significant by day 30. An increase in plasma osmolality and serum sodium concentration by pecavaptan was observed at day 7 and day 30 (both *P* < 0.01).

**Conclusions:**

In patients hospitalized for acute HF with residual congestion, pecavaptan showed modest signals of early decongestion, without durable benefit at 30 days. These post hoc findings require prospective confirmation. (A Trial to Study BAY1753011 in Patients With Congestive Heart Failure [AVANTI]; NCT03901729)

Currently, the only guideline-recommended therapy of acute decompensated heart failure (ADHF) remains decongestion using loop diuretics.^1^^,^^2^ However, the use of loop diuretics is complicated by electrolyte disturbances, worsening renal function, hypotension, and paradoxical arginine vasopressin (AVP) stimulation.^1^^,^^3^ These limitations are thought to contribute to the high prevalence of residual congestion at discharge, which is related to significantly worse outcomes.^4^

AVP, also known as antidiuretic hormone, may be a promising target as it is upregulated in heart failure (HF) and plays a central role in water retention and vascular tone through activation of the V2 and V1a-receptors.^5^, ^6^, ^7^ V2-receptor stimulation enhances water reabsorption in the renal collecting ducts via aquaporin-2, leading to hyponatremia, whereas V1a-receptor activation increases peripheral vascular resistance and may drive pathological cardiac remodeling through angiotensin II–like pathways.^5^, ^6^, ^7^

Previous trials with V2-receptor antagonists, including AQUAMARINE, EVEREST, TACTICS-HF, ACTIV in CHF, and SECRET of CHF all demonstrated significantly increased decongestive effects during hospitalization.^8^, ^9^, ^10^, ^11^, ^12^ However, these effects have not translated into reductions in HF rehospitalization or mortality.^5^

Dual V1a/V2-receptor antagonism has been hypothesized to offer broader benefits by not only enhancing decongestion but also mitigating hemodynamic stress and maladaptive remodeling through V1a blockade.^13^^,^^14^ Consequently, pecavaptan, a dual V1a/V2-receptor antagonist, was developed to overcome the limitations of existing V2-receptor antagonists. The AVANTI trial evaluated pecavaptan in patients hospitalized for ADHF with persistent congestion despite loop diuretic therapy. In this trial, pecavaptan did not significantly improve the primary endpoint of weight loss at 30 days.^15^ However, earlier studies suggest that the effects of AVP antagonism may predominantly occur early after initiation of therapy in patients admitted for worsening HF. For example, the AQUAMARINE trial found that effects of tolvaptan, a V2 receptor antagonist, on weight loss were more pronounced at 24 hours compared to 48 hours.^11^ Furthermore, both SECRET of HF and EVEREST trials showed that the effect of tolvaptan on weight loss (day 1 in SECRET of HF) and/or dyspnea (12 hours in EVEREST) was greatest in the short term.^10^^,^^12^ The 30-day weight change investigated in the main trial may be confounded by postdischarge factors such as increased fluid intake, potentially diluting early treatment effects. Accordingly, pecavaptan may have greater effects on short-term congestion. Therefore, in the present study, we examined whether the use of pecavaptan had a significant effect on shorter-term/7-day measures of decongestion and weight loss.

## Methods

### Study population

This is a post hoc, nonprespecified analysis of the AVANTI trial. AVANTI was a multicenter, randomized, double-blind, parallel group, active and placebo-controlled trial.^15^ Part A evaluated the efficacy and safety of adding 30 mg of oral pecavaptan to the standard-of-care therapy (including loop diuretics) compared to standard-of-care plus oral placebo over 30 days. In brief, patients were included in part A if they displayed at least 1 of the following 5 markers consistent with incomplete decongestion, despite initial intravenous loop diuretic therapy over 3 to 7 days: 1) persistent natriuretic peptide elevation, defined as brain natriuretic peptide (BNP) ≥500 pg/mL or N-terminal-pro-BNP(NT-proBNP) ≥1,800 pg/mL at screening, or a reduction of BNP or NT-proBNP by ≤30% from admission levels; 2) inadequate weight loss despite diuretic use by day 4 of the index hospitalization, defined as < 0.4 kg lost per 40 mg of furosemide; 3) a clinical composite congestion score (CCS) of ≥3, based on the severity of orthopnea, jugular venous pressure (JVP), and pedal edema; 4) hypervolemic hyponatremia; or 5) worsening kidney function during hospitalization, defined as an increase in serum creatinine of ≥0.3 mg/dL from admission levels, accompanied by signs of venous congestion, including at least 1 of the following: JVP ≥10 cm; inferior vena cava diameter >21 mm with <50% collapse on sniff; moderate to severe peripheral edema; or evidence of pulmonary edema/pleural effusion on X-ray or clinical examination.

Informed consent was obtained from all participants. The study was conducted in accordance with the Declaration of Helsinki and approved by the relevant institutional ethics committee. Details about the design of the study are available elsewhere.^16^

### Congestion assessments

Individual congestion parameters were graded into 4 categories at baseline, day 7, and day 30 as follows: orthopnea was scored from 0 (none) to 3 (continuous); JVP was graded from 0 (<6 cm H_2_O) to 3 (>15 cm H_2_O); and pedal edema was graded from 0 (absent or trace) to 3 (marked). Details on each category can be found in Supplemental Table 1. A CCS included the severity of orthopnea, JVP, and pedal edema, yielding a total score ranging from 0 to 9 (Supplemental Table 1).

### Biomarker measurements

All biomarkers were measured at baseline, day 7, and day 30. Soluble ST2 (sST2) was measured in plasma using the Human Luminex Discovery Assay, a multiplex immunoassay that detects proteins using antibody-coated magnetic microparticles. The assay was run on a Luminex instrument, which quantified biomarker concentrations based on fluorescence signals. Carbohydrate antigen 125 (CA125) was measured using a chemiluminescent microparticle immunoassay using the ARCHITECT CA125 II assay (lot 81007M800) (Abbott Laboratories).

### Outcomes

In this post hoc analysis, the primary outcomes were the effects of pecavaptan on weight change and CCS from baseline to day 7.

Exploratory outcomes included changes in symptom severity of pedal edema, orthopnea, and JVP, as well as thoracic fluid content, which was measured noninvasively using the ReDS device (Sensible Medical). Additional exploratory outcomes were systolic and diastolic blood pressure, NT-proBNP, CA125, sST2, serum sodium, plasma copeptin, and plasma osmolality. Serum osmolality was calculated at each time point using the formula: osmolality (mOsm/kg) = 2 × [Na^+^] + BUN/2.8 + Glucose/18, where [Na^+^] is the serum sodium concentration (mmol/L), BUN is blood urea nitrogen (mg/dL), and Glucose is serum glucose (mg/dL).^17^ Analyses were based on all 482 patients included in part A of the AVANTI study, with missing data handled on an outcome-by-outcome basis using complete case analysis, given that missingness was considered potentially related to clinical status and therefore not at random. Clinical characteristics of patients with available vs missing 7-day weight assessments are presented in Supplemental Table 2. All outcomes were assessed at baseline (predose), day 7 (post-treatment), and as exploratory analysis at day 30 if not previously reported.

### Statistical analysis

To estimate the absolute treatment effect of pecavaptan on change in weight and continuous outcomes at day 7, analysis of covariance adjusting for baseline values was used. Estimated marginal means with 95% CIs for each treatment group at day 7 were calculated using the emmeans package.^18^ Given the randomized design and demonstrated balance at baseline between treatment arms,^15^ each outcome was adjusted only for its own baseline value. The normality assumption was evaluated for each outcome/variable at baseline and day 7 using Q-Q plots of the residuals, and if necessary, variables were log-transformed to reduce skewness. This analysis assumed that the relationship between the change in outcome and their baseline values are linear, and that observations are independent of each other.

Changes in congestion symptom severity from baseline to day 7 were analyzed using a paired Wilcoxon signed-rank test to assess symptom improvement across all patients, irrespective of treatment. To investigate changes in individual parameters of the CCS (pedal edema, orthopnea, and JVP) over time, a cumulative link model was used, adjusting for baseline severity using the clm function from the “ordinal” package, as congestion parameters were assumed to be ordinal, rather than continuous.^19^ The proportional odds assumption was verified for the treatment effect in all models using nominal tests. Furthermore, a sensitivity analysis was performed using Generalized Estimating Equations (GEE) to account for the longitudinal and ordinal nature of the data to model symptom severity, fitted with the multgee package in R. A cumulative logit link function was specified with a row-column local OR structure to adjust for within-subject correlations across visits. The model included fixed effects for treatment, visit, and baseline symptom severity, along with interaction terms for treatment-by-visit and baseline-by-visit. An additional sensitivity analyses used cumulative link mixed-effects models for each congestion parameter at day 7 and day 30, including treatment, visit, treatment × visit interaction, baseline values, and baseline value × visit interaction, with a random intercept for each patient.

Although this analysis focuses on early decongestion at day 7, we also performed exploratory analyses of congestion parameters through day 30. For all exploratory outcomes, the Benjamini-Hochberg procedure was additionally applied to control the false discovery rate, separately for each model type (analysis of covariance and ordinal regression) and time point. False discovery rate-adjusted *P* values are provided in all tables and figures; findings not surviving correction are noted in the text.

## Results

### Patient characteristics

The median age of patients was 70 years (IQR: 62.0; 78.0), 24% (118/482) were women, and 61% (293/479) had an ejection fraction <40%. At baseline, patients had a median CCS score of 3 (IQR: 2.00-4.00), a median weight of 82.8 kg (IQR: 72.3-94.9), and a median NT-proBNP concentration of 2020 pg/mL (IQR: 928-4,240). Further details on baseline characteristics of the AVANTI cohort have been published previously.^15^

### Effect of pecavaptan on body weight and the composite congestion score at day 7

**Figure 1:**
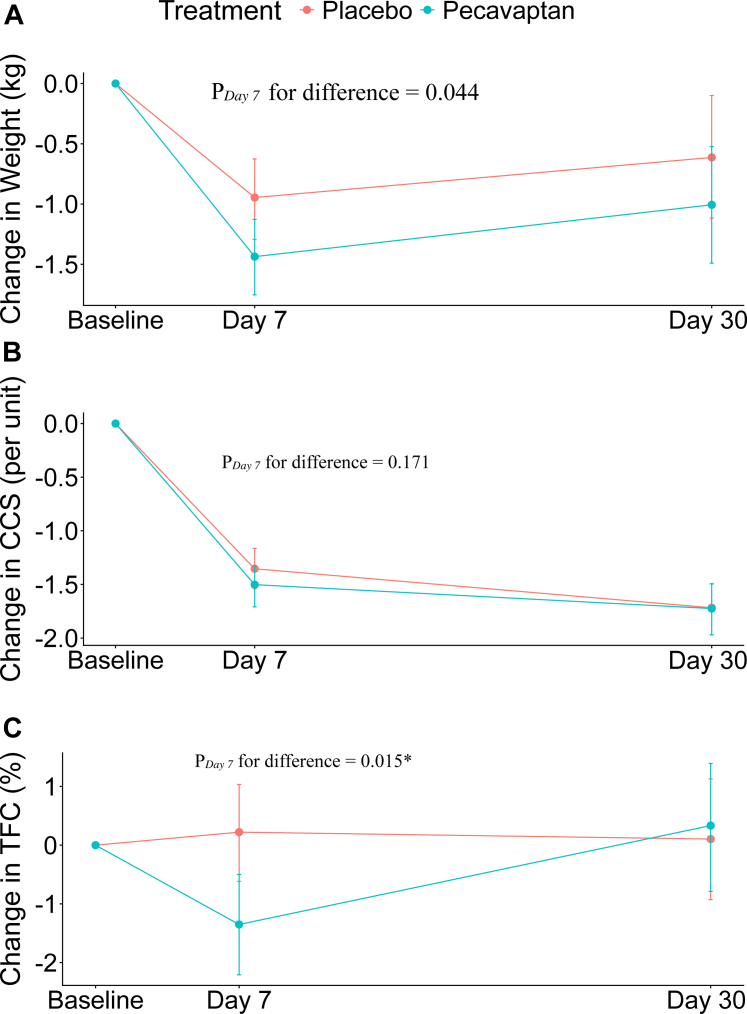
Trajectories On Congestion Parameters Over Time By Treatment Group Trajectories of A) weight loss, B) composite congestion scores, and C) thoracic fluid content over time. P for difference at day 7 was analyzed using ANCOVA, adjusting for baseline measurements. ∗The effect of Pecavaptan on TFC remained significant after false discovery rate correction using the Benjamini-Hochberg procedure. Error bars represent 95% bootstrap CIs. CCS = composite congestion score; TFC = thoracic fluid content.

When adjusting for baseline weight, weight loss at day 7 was −0.95 kg (95% CI: −1.33 to −0.58) in the placebo group vs −1.43 kg (95% CI: −1.80 to −1.05) in the pecavaptan group (difference: −0.47 kg; 95% CI: −0.94, −0.01; *P* = 0.044) (Table 1, Figure 1).

**Table 1:** Effects of Pecavaptan on Congestion Outcomes on Day 7

Endpoints	Placebo + SoC (n = 240)	Pecavaptan 30 mg + SoC (n = 242)	EM Mean Treatment Difference Between Groups (95% CI); 2-Sided *P* Value	Sample Size
Primary endpoint				
Body weight (kg) change, EM mean (95% CI)	−0.95 (−1.33 to −0.58)	−1.43 (−1.80 to −1.05)	−0.47 (−0.94, −0.01); ***P* = 0.044**	456
CCS change, EM mean (95% CI)	−1.35 (−1.54 to −1.16)	−1.51 (−1.70 to −1.32)	−0.16 (−0.40, 0.07); *P* = 0.171	453
Exploratory endpoints				
Thoracic fluid content change %, EM mean (95% CI)	0.15 (−0.79 to 1.08)	−1.28 (−2.20 to −0.36)	−1.43 (−2.57, −0.28); ***P* = 0.015, adj-*P* = 0.033**	303
Log-NT-proBNP change (pg/mL), EM mean (95% CI)	−0.02 (−0.11 to 0.07)	−0.04 (−0.13 to 0.05)	−0.02 (−0.13, 0.09); *P* = 0.737, adj-*P* = 0.760	417
Log-CA125 change (U/mL), EM mean (95% CI)	−0.28 (−0.39 to −0.17)	−0.22 (−0.34 to −0.10)	0.06 (−0.08, 0.20); *P* = 0.377, adj-*P* = 0.565	158
Log-sST2 change (pg/mL), EM mean (95% CI)	−0.12 (−0.19 to −0.04)	−0.09 (−0.17 to −0.01)	0.02 (−0.07, 0.12); *P* = 0.625, adj-*P* = 0.760	250
Plasma osmolality change (mOsmol/kg), EM mean (95% CI)	−2.05 (−4.58 to 0.48)	2.98 (0.37-5.58)	5.02 (1.85, 8.20); ***P* = 0.002, adj-*P* = 0.006**	394
Plasma log-copeptin change (pmol/L), EM mean (95% CI)	−0.06 (−0.13 to 0.02)	0.54 (0.46-0.62)	0.60 (0.50-0.69); ***P* < 0.001, adj-*P* < 0.001**	452
Plasma sodium change (mmol/L), EM mean (95% CI)	−1.13 (−2.29 to 0.03)	1.41 (0.23-2.60)	2.54 (1.09, 3.99); ***P* < 0.001, adj-*P* = 0.003**	418
Systolic blood pressure change (mm Hg), EM mean (95% CI)	0.77 (−1.50 to 3.05)	1.21 (−1.06 to 3.48)	0.44 (−2.37, 3.25); *P* = 0.760, adj-*P* = 0.760	443
Diastolic blood pressure change (mm Hg), EM mean (95% CI)	0.57 (−0.96 to 2.10)	1.50 (−0.02 to 3.03)	0.94 (−0.95, 2.82); *P* = 0.331, adj-*P* = 0.565	443

Estimated marginal (EM) means were calculated using analysis of covariance (ANCOVA), adjusting for baseline values. EM mean treatment differences were calculated using post hoc testing in a pairwise manner.

Exploratory endpoints were adjusted for multiple testing using the Benjamini-Hochberg procedure (adj-P). **Bold** values are highlighting the significant *P* values.

CA125 = carbohydrate antigen 125; CCS = composite congestion score; NT-proBNP = N-terminal pro-B-type natriuretic peptide; SoC = standard of care; sST2 = soluble ST2.

CCS scores decreased significantly in both groups over 7 days (*P* < 0.001). After adjusting for baseline CCS, the CCS score decreased by −1.35 (95% CI: −1.54 to −1.16) in the placebo group and −1.51 (95% CI: −1.70 to −1.32) in the pecavaptan group (difference: −0.16; 95% CI: −0.40 to 0.07; *P* = 0.171) (Figure 1, Table 1).

### Effect on individual parameters of congestion

**Figure 2:**
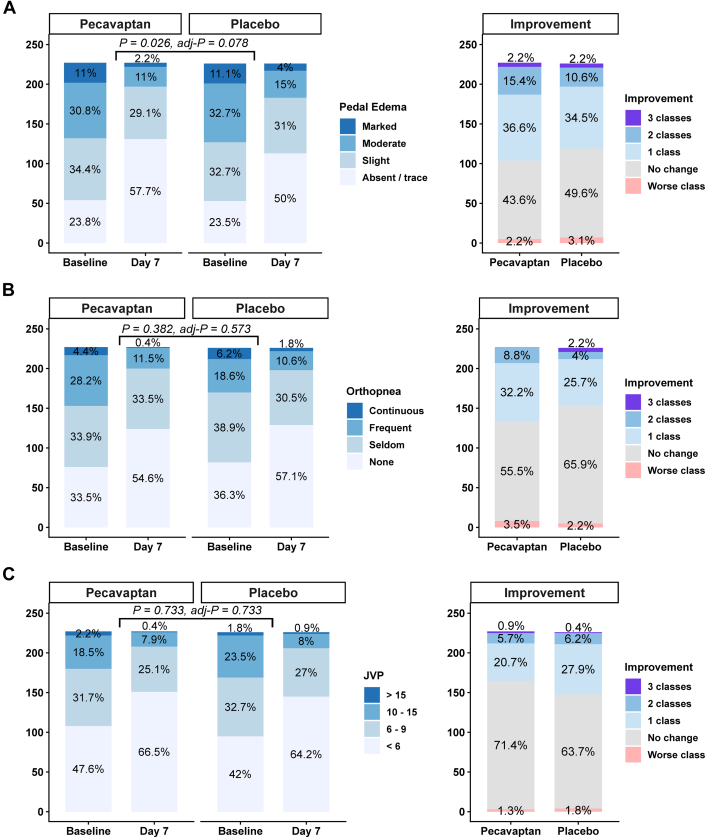
Congestion Severity Over Time A) Effect on pedal edema, B) effect on orthopnea, and C) effect on jugular venous pressure (JVP). *P* value for difference was calculated via ordinal regression, predicting symptoms severity at day 7, adjusting for baseline symptom severity. Only patients with both baseline and day 7 measurements are included. Adjusted *P* values were adjusted using the Benjamini-Hochberg procedure. Twenty-nine patients had missing pedal edema, orthopnea, and/or JVP assessments at baseline or day 7.

**Figure 3:**
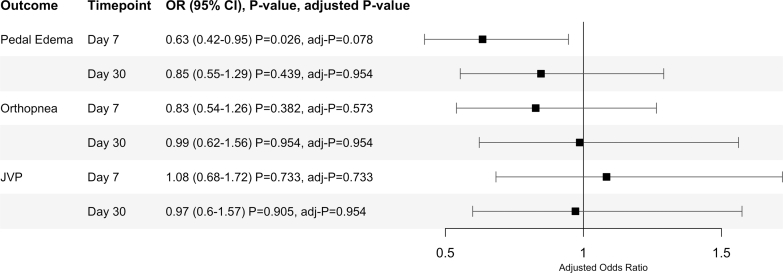
The Relationship Between Pecavaptan Treatment and Congestion Severity at Day 7 and Day 30, Adjusted for Baseline Severity Calculated using ordinal regression, correcting for baseline congestion severity. ORs and CIs are reported. Adjusted *P* values were adjusted using the Benjamini-Hochberg procedure. JVP = jugular venous pressure.

Clinical signs and symptoms of congestion, including pedal edema, orthopnea, and elevated JVP, improved significantly in both groups over 7 days (all *P* < 0.001). By day 7, the proportion of patients with absent/trace edema was 58% (131/227) in the pecavaptan group vs 50% (113/226) in the placebo group, with 18% (40/227) improving 2 classes or more in the pecavaptan group vs 13% (29/226) in the placebo group (Figure 2A). At day 7, those treated with pecavaptan had 0.63 lower odds of being in a higher symptom severity category compared to placebo after adjusting for baseline severity (adjusted-OR: 0.63; 95% CI: 0.42-0.95; nominal *P* = 0.026) (Figure 3). This finding did not survive correction for multiple comparisons (Benjamini-Hochberg-adjusted *P* = 0.078). By day 30, the observed benefits of pecavaptan on pedal edema were no longer significant. The sensitivity analysis using GEE found similar results (Supplemental Table 3).

In contrast, pecavaptan had no significant effect on orthopnea or JVP in either the baseline-adjusted ordinal regression or GEE analysis (Figure 3). Furthermore, there was no effect on systolic or diastolic blood pressure (Table 1). However, patients treated with pecavaptan also showed a greater reduction in thoracic fluid content compared to placebo at day 7 (pecavaptan: −1.28%; placebo: +0.15%; between-group difference: −1.43%; 95% CI: −2.57 to −0.28; *P* = 0.015) (Table 1). The results of the cumulative link mixed model analysis on congestion parameters can be found in Supplemental Table 4.

### Effects on plasma osmolality, NT-proBNP, CA125, and sST2

**Figure 4:**
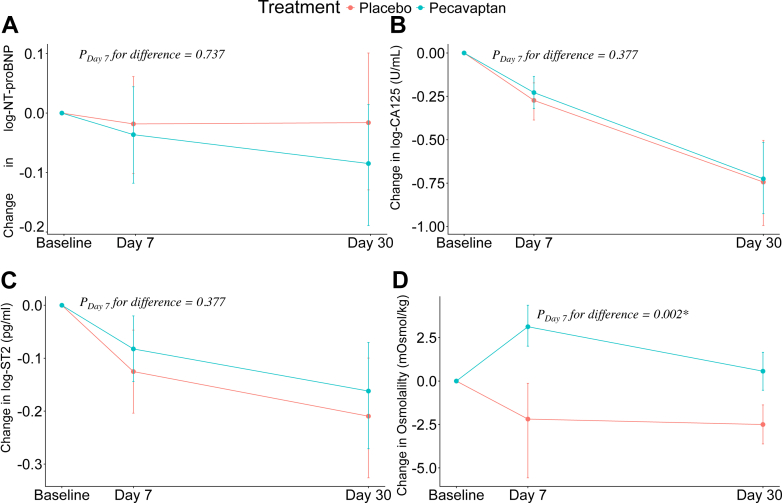
Biomarker Trajectories Over Time in Pecavaptan vs Placebo A) NT-proBNP, B) CA125, C) sST2, and D) plasma osmolality. *P* for difference at day 7 was analyzed using ANCOVA, adjusting for baseline measurements. ∗The effect of Pecavaptan on plasma osmolality remained significant after false discovery rate correction using the Benjamini-Hochberg procedure. Error bars represent 95% bootstrap CIs. CA125 = cancer antigen 125; NT-proBNP = N-terminal pro-B-type natriuretic peptide; sST2 = soluble ST2.

Pecavaptan significantly increased plasma osmolality, and serum sodium concentrations over 7 days compared to placebo (*P* < 0.01) (Table 1), and these effects were sustained through day 30 (Figure 4, Supplemental Table 5). Although NT-proBNP, CA125, and sST2 levels decreased in all patients over 7 days, there were no differences between treatment groups (Table 1, Figure 4). Lastly, plasma copeptin levels were increased at both 7 and 30 days in response to pecavaptan therapy (*P* < 0.001) (Table 1, Supplemental Table 5).

**Central Illustration:**
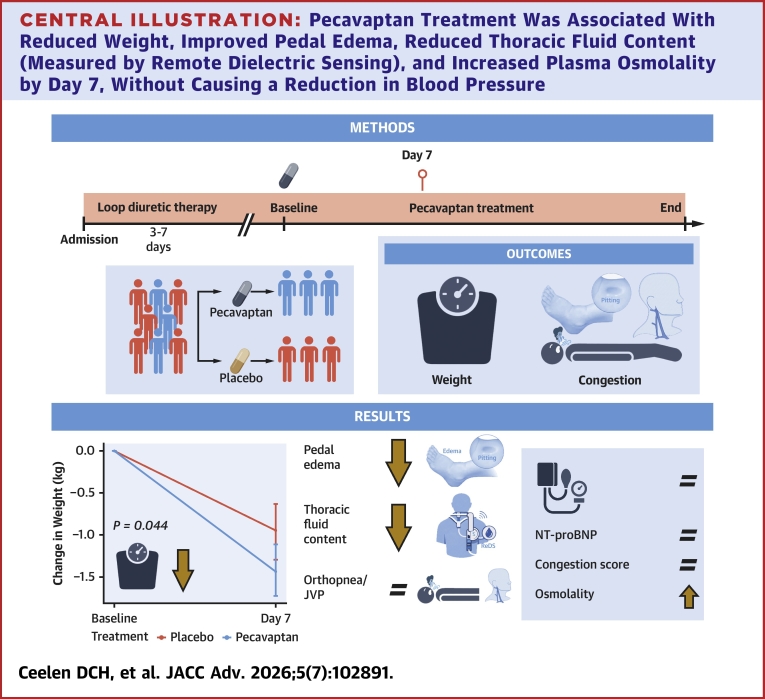
Pecavaptan Treatment Was Associated With Reduced Weight, Improved Pedal Edema, Reduced Thoracic Fluid Content (Measured by Remote Dielectric Sensing), and Increased Plasma Osmolality by Day 7, Without Causing a Reduction in Blood Pressure NT-proBNP = N-terminal pro-B-type natriuretic peptide; JVP = jugular venous pressure.

## Discussion

In this post hoc analysis of a randomized, double-blinded study of patients hospitalized for ADHF with residual congestion, pecavaptan modestly improved decongestion during the first 7 days. Pecavaptan treatment was associated with reduced weight, nominally improved pedal edema and increased plasma osmolality and sodium concentrations by day 7, without causing a reduction in blood pressure. However, these effects were not accompanied by changes in biomarkers of congestion and not sustained at day 30, suggesting that although pecavaptan may offer modest short-term decongestion benefits, the long-term clinical impact may be limited.

These findings align with prior studies of vasopressin antagonists. The EVEREST trial showed that tolvaptan reduced body weight and edema within the first days of administration.^20^ Similarly, the ACTIV-CHF and SECRET of CHF trials found reduced weight in those treated with tolvaptan.^8^^,^^10^ Finally, AQUAMARINE, an open label trial investigating early tolvaptan administration, demonstrated that tolvaptan increased weight loss and fluid loss within 48 hours.^11^

### Effect on signs and symptoms of congestion

Pecavaptan improved several markers of tissue congestion including weight, pedal edema, and thoracic fluid content, without significantly affecting blood pressure. However, the absence of meaningful effects on markers of intravascular congestion such as orthopnea and JVP resulted in the overall effect on the CCS at day 7 being neutral. The rise in plasma osmolality and sodium levels after pecavaptan treatment suggests enhanced aquaresis, which may have facilitated interstitial fluid mobilization. These effects on sodium concentration are consistent with prior trials of tolvaptan.^8^^,^^20^ The enhanced fluid mobilization may, in turn, explain the observed reductions in pedal edema and thoracic fluid content. Although this proposed mechanism remains speculative, it warrants further investigation. Notably, although the increases in plasma osmolality and serum sodium persisted through 30 days, the effects on body weight, thoracic fluid content, and pedal edema did not. This decoupling indicates that improved fluid mobilization by increased osmolality alone may not fully account for the observed clinical improvements as the decongestive effects were not maintained through day 30.

The absence of a blood pressure–lowering effect with pecavaptan is consistent with findings from the AQUAMARINE trial, the ACTIV-CHF trial and a pilot study of conivaptan (another dual V1a/V2-antagonist).^8^^,^^11^^,^^21^ This is reassuring, as the addition of V1a receptor blockade does not appear to induce hypotension, a common and clinically significant barrier to effective decongestive therapy, and a predictor of adverse outcomes in patients with ADHF.^22^ However, we found that the beneficial effects of pecavaptan on weight loss and other signs of congestion at day 7 were not apparent anymore at day 30. This is in contrast with findings of EVEREST and ACTIV-CHF, which showed a sustained reduction in body weight over time.^8^^,^^20^ Furthermore, the weight loss observed in this trial (−0.47 kg at 7 days post-treatment) was more modest than in EVEREST (−0.79 kg at 1 day post-treatment), which may be partly explained by timing: patients in AVANTI were enrolled later in their clinical course, after conventional loop diuretic therapy had already produced weight reduction.^20^ Furthermore, <13% of patients were included based on insufficient weight loss at day 3 to 7 postadmission, and only 25% had a CCS of 3 or more at screening, leaving less room for vasopressin antagonism to add further benefit.^15^ Although the weight reduction reached statistical significance, the absolute magnitude (−0.47 kg) is unlikely to be clinically meaningful on its own; however, the concurrent changes in pedal edema and thoracic fluid content may reflect a modest physiological signal.

### Possible mechanisms that abolish the long-term effect of pecavaptan

The mechanism underlying the lack of sustained benefit remains speculative. Firstly, the relatively late initiation of pecavaptan in our study (3-7 days postadmission) may have attenuated its clinical impact, although prior data on tolvaptan suggest some benefits of vaptans even when introduced later during hospitalization.^12^ Another potential mechanism may involve compensatory increases in circulating AVP following V1a/V2R blockade, as reflected by the observed rise in copeptin levels after treatment in this study. This may stimulate V1b (also known as V3) receptor signaling, causing adrenocorticotropic hormone release and, downstream, elevation of renin, and aldosterone in the long term.^23^ Perhaps more likely, sustained aquaresis may induce mild hypovolemia triggering renin-angiotensin-aldosterone system activation and possibly promoting structural renal adaptations, such as proximal tubular hypertrophy, further limiting decongestive efficacy.^24^ Consistently, AVANTI observed increased aldosterone at day 30, contrasting with prior short-term vasopressin antagonism studies that reported minimal neurohumoral changes.^15^^,^^25^^,^^26^

An alternative mechanism is that increased plasma osmolality may increase thirst by stimulating the central osmoreceptors,^27^ promoting fluid intake that counteracts the aquaretic benefit over time. Notably, thirst and polydipsia are one of the most reported side-effects of tolvaptan use,^28^ and an increased fluid intake was observed with tolvaptan in AQUAMARINE.^11^ These observations suggest the hypothesis that vasopressin antagonists may provide short-term benefit during the in-hospital phase, when fluid restriction is standard practice, but that this benefit may be attenuated postdischarge due to compensatory neurohumoral changes and increased fluid intake. However, these mechanisms are outside the scope of this paper and warrant further studies to elucidate long-term adaptations and to delineate the chronic renal and neurohumoral consequences of dual vasopressin receptor antagonism.

From a clinical standpoint, these findings highlight the possible utility of pecavaptan as an adjunctive therapy to potentially accelerate decongestion in patients with residual congestion during hospitalization. Even in the absence of sustained effects, early relief of symptoms and volume overload may modestly improve in-hospital management and patient comfort. However, as these analyses are post hoc in nature and this was a neutral trial, our results should be considered hypothesis-generating only and require prospective validation in dedicated trials. The role of dual V1a/V2 antagonism in targeting (tissue) congestion without adversely affecting hemodynamics requires further mechanistic investigation. Longer-term studies are necessary to assess whether short-term improvements in decongestion results in better postdischarge outcomes and to identify the compensatory neurohumoral mechanisms limiting sustained efficacy. Furthermore, future studies could be useful in patients with limitations to the use of diuretics, especially those with either Stage 3 or 4 chronic kidney disease or those who develop worsening renal function during early decongestion.

### Study Limitations

This study had several limitations. First, the large majority of patients were included based on consistently elevated natriuretic peptide levels, rather than insufficient weight loss or residual signs/symptoms of congestion. This selection bias could explain the limited effects of weight loss and congestion parameters observed in the trial, as natriuretic peptides may reflect elevated pressure/low output, rather than (residual) intravascular congestion alone. Moreover, this bias may account for the lack of impact on biomarkers, as NT-proBNP and CA125 levels were paradoxically higher in those without clinical congestion who were included based on elevated natriuretic peptide levels. Second, although multiple endpoints suggested improved decongestion, the study had limited power to detect effects on exploratory outcomes and was underpowered to detect differences in long-term clinical outcomes, such as mortality or rehospitalization. Furthermore, the interpretation of long-term effects is complicated by the study design: in part B, participants in each treatment arm were re-randomized into 3 additional arms, introducing heterogeneity that limits conclusions beyond the initial phase. Complete case analyses were performed on an outcome-by-outcome basis; as patients with missing data had higher illness severity at baseline, findings may not be fully generalizable to the sickest patients in the cohort. Lastly, the observed reduction in thoracic fluid content of 1.43% should be interpreted with caution; for context, prior data have demonstrated a mean thoracic fluid content reduction of approximately 4.7% associated with clinical decongestion sufficient for discharge, suggesting the magnitude observed here is of uncertain clinical significance.^29^

## Conclusions

In patients hospitalized for acute HF with residual congestion, pecavaptan modestly improved signs and symptoms of congestion and increased weight loss. However, these beneficial effects were no longer evident at 30 days. These exploratory findings are hypothesis-generating and suggest a potential short-term benefit in reducing tissue congestion. Prospective trials with prespecified short-term endpoints are needed before further conclusions can be drawn.

Perspectives **COMPETENCY IN MEDICAL KNOWLEDGE:** Pecavaptan facilitated modest decongestion during the initial 7 days of therapy, evidenced by weight loss, resolution of peripheral edema, and increases in plasma osmolality and sodium concentrations. Notably, these effects occurred without reducing systemic blood pressure. However, these effects were not sustained at day 30, suggesting that although pecavaptan may offer modest short-term decongestion benefits, the long-term clinical impact may be limited.

**TRANSLATIONAL OUTLOOK:** Building on the short-term efficacy and safety profile observed in this study, further studies could be useful in patients with limitations to the use or efficacy of diuretics. For example, patients with stage 3 or 4 chronic kidney disease and those who develop worsening renal function during conventional decongestive therapy.

### Declaration of generative AI and AI-assisted technologies in the writing process

AI programs were used for proof reading and the creation of an icon in the Central Illustration.

## Funding support and author disclosures

The trial was funded by 10.13039/100004326Bayer AG, Wuppertal, Germany. The employer of Dr Voors received consultancy fees and/or research support from Adrenomed, Anacardio, Armgo, 10.13039/100004325AstraZeneca, Bayer AG, BMS, Boehringer Ingelheim, Cardurion, Corteria, EliLilly, Merck, Moderna, Novartis, 10.13039/501100004191Novo Nordisk, and SalubrisBio. Dr Frost is an employee of Bayer AG. Dr ter Maaten is supported by a Veni grant from 10.13039/501100001826ZonMW; has received consulting and/or speaker fees to institution from Bayer, Boehringer Ingelheim, Moderna, Novartis, Novo Nordisk, Roche, and Johnson and Johnson; and served on the data safety monitoring board for FASTR. Dr Gustafsson is a full-time employee of Abbott. Dr Monzo has received a travel grant from 10.13039/100001003Boehringer Ingelheim. All other authors have reported that they have no relationships relevant to the contents of this paper to disclose.
