# Supplementary material for: Short-Term Effects of Pecavaptan on Weight and Congestion in Acute Heart Failure: The AVANTI Post-Hoc Analysis
Source: JACC Adv. 2026 Jul 22;5(7):102891. doi: 10.1016/j.jacadv.2026.102891 (PMC13400116; doi:10.1016/j.jacadv.2026.102891)
Supplement: Supplementary Tables 1 to 5 [file mmc1.docx]

**SUPPLEMENTAL MATERIALS**

**Supplemental Table 1. Grading of clinical composite congestion score (CCS) and congestion assessment classications.**

| **Signs/Symptoms** | **0** | **1** | **2** | **3** |
| --- | --- | --- | --- | --- |
| Orthopnoea | None | Seldom | Frequent | Continuous |
| JVP (cm H_2_O) | <6 | 6-9 | 10-15 | >15 |
| Pedal oedema | Absent/trace | Slight | Moderate | Marked |

*Jugular venous pressure (JVP).*

**Supplemental Table 2. Baseline characteristics of patients with missing weight at day 7 versus those with available weight data at day 7.**

|  | **Available weight at day 7 N=381** | **Missing weight at day 7 N=101** | **p.overall** | **N** |
| --- | --- | --- | --- | --- |
| Age (years) | 70.0 [62.0;77.0] | 72.0 [65.0;79.0] | 0.091 | 482 |
| Female sex, n(%): | 88 (23.1%) | 30 (29.7%) | 0.214 | 482 |
| White race, n(%): | 378 (99.2%) | 101 (100%) | 1.000 | 482 |
| Randomized to Pecavaptan, n(%): | 191 (50.1%) | 51 (50.5%) | 1.000 | 482 |
| LVEF < 40%: | 232 (61.2%) | 61 (61.0%) | 1.000 | 479 |
| Weight at Baseline (kg) | 83.4 [72.9;95.0] | 78.9 [70.9;91.2] | 0.105 | 478 |
| BMI (kg/m²) | 29.1 [25.3;32.6] | 28.4 [25.4;31.7] | 0.440 | 477 |
| Systolic Blood Pressure (mmHg) | 118 [108;130] | 115 [104;127] | 0.095 | 469 |
| Diastolic Blood Pressure (mmHg) | 69.0 [62.0;76.0] | 67.5 [61.0;76.5] | 0.418 | 469 |
| Heart Rate (bpm) | 69.0 [60.5;77.5] | 69.2 [60.5;77.5] | 0.700 | 469 |
| Congestion Score (Baseline) | 3.00 [2.00;4.00] | 3.00 [2.00;5.00] | 0.016 | 482 |
| NYHA Class, n(%): |  |  | 0.162 | 482 |
| I | 5 (1.31%) | 0 (0.00%) |  |  |
| II | 119 (31.2%) | 25 (24.8%) |  |  |
| III | 245 (64.3%) | 69 (68.3%) |  |  |
| IV | 12 (3.15%) | 7 (6.93%) |  |  |
| History of Myocardial Infarction, n(%): | 30 (7.87%) | 7 (6.93%) | 0.915 | 482 |
| Diabetes Mellitus, n(%): | 195 (51.2%) | 49 (48.5%) | 0.715 | 482 |
| Hypertension, n(%): | 308 (80.8%) | 81 (80.2%) | 0.997 | 482 |
| Chronic Kidney Disease, n(%): | 145 (38.1%) | 55 (54.5%) | 0.004 | 482 |
| **Medication use** |  |  |  |  |
| RAAS Inhibitors, n(%): | 320 (84.0%) | 79 (78.2%) | 0.223 | 482 |
| Beta-blockers, n(%): | 346 (90.8%) | 90 (89.1%) | 0.743 | 482 |
| Calcium Channel Blockers, n(%): | 68 (17.8%) | 23 (22.8%) | 0.326 | 482 |
| Aldosterone Antagonists, n(%): | 279 (73.2%) | 78 (77.2%) | 0.492 | 482 |
| Thiazide Diuretics, n(%): | 60 (15.7%) | 20 (19.8%) | 0.410 | 482 |
| SGLT2 Inhibitors, n(%): | 65 (17.1%) | 10 (9.90%) | 0.107 | 482 |
| **Laboratory values** |  |  |  |  |
| eGFR (mL/min/1.73m²) | 54.2 [43.0;69.2] | 45.7 [36.3;60.3] | 0.001 | 438 |
| Serum Sodium (mmol/L) | 140 [138;142] | 140 [138;142] | 0.490 | 456 |
| Serum Potassium (mmol/L) | 4.30 [4.00;4.60] | 4.30 [4.10;4.65] | 0.834 | 455 |
| NT-proBNP at Baseline (pg/mL) | 1877 [772;4027] | 2940 [1218;5648] | 0.002 | 456 |

*Data are presented as mean (SD), median (quartiles), or n (%). Abbreviations: ACE, angiotensin-converting enzyme; ARB, angiotensin II receptor blocker; eGFR, estimated glomerular filtration rate; LVEF, left ventricular ejection fraction; NT-proBNP, N-terminal pro B-type natriuretic peptide; NYHA, New York Heart Association; RAAS, renin-angiotensin-aldosterone system; SGLT2, sodium-glucose cotransporter 2.*

**Supplemental Table 3. Longitudinal Analysis of Clinical Signs of Congestion.**

| **Endpoint** | **OR for effect at day 7** | **P value** | **OR for time (day 30 x Treatment)** | **P for intearction** |
| --- | --- | --- | --- | --- |
| Peripheral Edema | 0.62 (0.42 to 0.93) | **0.022** | 1.38 (0.92 to 2.09) | 0.123 |
| JVP | 1.20 (0.75 to 1.90) | 0.449 | 0.80 (0.52 to 1.22) | 0.295 |
| Orthopnea | 0.83 (0.55 to 1.26) | 0.393 | 1.09 (0.69 to 1.72) | 0.711 |

*Treatment effects were estimated using proportional odds GEE (Generalized Estimating Equations) models with a cumulative logit link function. This approach models the odds of being in a lower (improved) category of symptom severity (e.g., transitioning from 'Severe' to 'Moderate' or 'None'). The models included Treatment group, Visit (Day 7 vs. Day 30), and their interaction as fixed effects, while adjusting for baseline symptom severity and the interaction of baseline with visit. To account for the within-subject correlation of repeated measures, a random-effects structure (RC - Row-Column) was utilized. Odds Ratios (OR) > 1.0 indicate higher odds of achieving a lower (better) symptom grade with Pecavaptan compared to Placebo. Statistical significance was determined using robust (sandwich) standard error.*

**Supplemental Table 4. Effects of Pecavaptan on individual congestion parameters over 30 days.**

| **Outcome** | **OR** | **Lower 95%CI** | **Upper  95%CI** | **P-value** |
| --- | --- | --- | --- | --- |
| **Pedal edema** |  |  |  |  |
| Effect of Pecavaptan | 0.45 | 0.23 | 0.90 | 0.024 |
| Interaction between pecavaptan and time | 1.83 | 0.90 | 3.71 | 0.093 |
| **Jugular Venous Pressure** |  |  |  |  |
| Effect of Pecavaptan | 1.40 | 0.43 | 4.52 | 0.577 |
| Interaction between pecavaptan and time | 0.75 | 0.28 | 1.99 | 0.562 |
| **Orthopnea** |  |  |  |  |
| Effect of Pecavaptan | 0.78 | 0.38 | 1.61 | 0.499 |
| Interaction between pecavaptan and time | 1.33 | 0.61 | 2.89 | 0.469 |

*Odds Ratio (OR) with 95% Confidence Interval (CI) is reported, adjusted for baseline symptom severity.*

**Supplemental Table 5. Effects of Pecavaptan on congestion outcomes on day 30**

| \|  \| **Placebo + SoC (N=240)** \| \| **Pecavaptan 30 mg + SoC (N=242)** \| **EM mean treatment difference between groups (95% CI); two-sided *P*-value** \| **Sample Size** \| \| --- \| --- \| --- \| --- \| --- \| --- \| \| CCS change, EM Mean (95% CI) \| -1.71 (-1.92 to -1.51) \| -1.73 (-1.93 to -1.53) \| \| -0.02 (-0.27 to 0.23), p=0.893 \| 395 \| \| **Exploratory endpoints** \|  \|  \| \|  \|  \| \| Log-CA125 change (U/mL), EM Mean (95% CI) \| -0.75 (-0.94 to -0.55) \| -0.72 (-0.92 to -0.53) \| \| 0.02 (-0.22 to 0.26); p=0.853, adj-P=0.853 \| 123 \| \| Log-sST2 change (pg/mL), EM Mean (95% CI) \| -0.20 (-0.30 to -0.10) \| -0.17 (-0.28 to -0.06) \| \| 0.03 (-0.10 to 0.16); P=0.615, adj-P=0.691 \| 220 \| \| Plasma osmolality change (mOsmol/kg), EM Mean (95% CI) \| -2.44 (-3.64 to -1.25) \| 0.51 (-0.66 to 1.68) \| \| 2.96 (1.50 to 4.42); **P<0.001, adj-P<0.001** \| 351 \| \| Plasma log-copeptin change (pmol/L), EM Mean (95% CI) \| -0.12 (-0.20 to -0.04) \| 0.47 (0.39 to 0.55) \| \| 0.59 (0.50 to 0.69); **P<0.001, adj-P<0.001** \| 389 \| \| Plasma sodium change (mmol/L), EM Mean (95% CI) \| -0.86 (-1.41 to -0.31) \| 0.75 (0.21 to 1.29), \| \| 1.61 (0.93 to 2.28); **P<0.001, adj-P<0.001** \| 375 \| \| Systolic blood pressure change (mmHg), EM Mean (95% CI) \| 4.25 (1.65 to 6.85) \| 3.34 (0.80 to 5.87) \| \| -0.91 (-4.09 to 2.26); P=0.572, adj-P=0.691 \| 383 \| \| Diastolic blood pressure change (mmHg), EM Mean (95% CI) \| 2.83 (1.07 to 4.59) \| 2.26 (0.54 to 3.98) \| \| -0.57 (-2.73 to 1.58); P=0.601, adj-P=0.691 \| 383 \| |
| --- | --- | --- | --- | --- | --- | --- | --- | --- | --- | --- | --- | --- | --- | --- | --- | --- | --- | --- | --- | --- | --- | --- | --- | --- | --- | --- | --- | --- | --- | --- | --- | --- | --- | --- | --- | --- | --- | --- | --- | --- | --- | --- | --- | --- | --- | --- | --- | --- | --- | --- | --- | --- | --- | --- | --- | --- | --- | --- | --- | --- |

*Estimated Marginal (EM) means were calculated using analysis of covariance (ANCOVA), adjusting for baseline values. Estimated marginal mean treatment differences were calculated using post-hoc testing in a pairwise manner, adjusting for multiple testing using the Benjamini-Hochberg method. CA125 (Carbohydrate Antigen 125), CI (Confidence Interval), Composite Congestion Score (CCS), NT-proBNP (N-terminal pro-B-type Natriuretic Peptide), SoC (Standard of Care), and sST2 (soluble ST2). Exploratory endpoints were adjusted for multiple testing using the Benjamini-Hochberg procedure.*
